# Timing of Cardiac Contractility Modulation Therapy Implementation

**DOI:** 10.1002/clc.70507

**Published:** 2026-10-31

**Authors:** Goekhan Yuecel, Gamze Yildirim, Kristin Schreiner, Svetlana Hetjens, Konstantinos Giannakopoulos, Abbass Kodeih, Mostafa Khalil, Babak Yazdani, Faeq Husain‐Syed, Daniel Duerschmied, William T. Abraham, Ibrahim Akin, Jürgen Kuschyk

**Affiliations:** ^1^ First Department of Medicine University Medical Centre Mannheim, Medical Faculty Mannheim of the University of Heidelberg Mannheim Germany; ^2^ German Centre for Cardiovascular Research, Partner Site Heidelberg‐Mannheim Mannheim Germany; ^3^ Department of Medical Statistics and Biomathematics University Medical Centre Mannheim, Medical Faculty Mannheim of the Heidelberg University Mannheim Germany; ^4^ First Department of Cardiology Hippokration General Hospital National and Kapodistrian University of Athens Athens Greece; ^5^ Fifth Department of Medicine University Medical Centre Mannheim, Medical Faculty Mannheim of the Heidelberg University Mannheim Germany; ^6^ Department of Internal Medicine II, Division of Nephrology University Hospital Giessen and Marburg Justus‐Liebig‐University Giessen Giessen Germany; ^7^ Division of Cardiovascular Medicine The Ohio State University Columbus Ohio USA

**Keywords:** cardiac contractility modulation, CIED, heart failure, treatment timing

## Abstract

**Aims:**

Symptomatic chronic heart failure (HF) despite medical treatment can be addressed with cardiac contractility modulation (CCM) therapy, which has yet to have been investigated regarding outcome differences between early versus late implementation.

**Methods and Results:**

CCM recipients from the Mannheim Cardiac Contractility Modulation Observational Registry (MAINTAINED) aged ≤ 70 years at implantation were grouped with regard to implantation time after the first diagnosis of CCM indicating HF (New York Heart Association classification (NYHA) ≥ 2 and left ventricular ejection fraction (LVEF) = 25%–45%) into either Early‐ (EC, *n* = 50, implantation < 13 months after diagnosis) or Late‐Implantation Group (LC, *n* = 81, implantation ≥ 13 months after diagnosis). Both groups were compared regarding survival, NYHA, LVEF, and HF‐related hospitalizations for up to 60 months. The mean implantation time after the first diagnosis were 5.85 ± 3.1 versus 92 ± 70.2 months (first values EC, *p* < 0.05). Mean survival was better for EC (55.2 ± 1.7 vs. 52.8 ± 1.6 months, *p* < 0.05). Improvements in NYHA (2.98 ± 0.47 to 2.0 ± 0.73 vs. 3.01 ± 0.46 to 2.25 ± 0.8, both *p* < 0.05) and LVEF (23.2 ± 7.5 to ± 38.82 ± 12.85 vs. 23.9 ± 7 to 29.92 ± 8.9%, each *p* < 0.05) were comparable, with differences in terms of grouping. Hospitalizations due to HF were more frequent to LC patients (39 vs. 110 events, Odds Ratio 2.1).

**Conclusions:**

Heart failure patients might experience improvements in functional status and left ventricular reverse remodeling following CCM therapy, regardless of the timing of implantation. Early implementation of CCM within the first year of CCM suitable HF might provide additional benefits regarding survival, left ventricular systolic recovery and protection against heart failure hospitalizations.

## Graphical abstract

In this retrospective registry analysis, cardiac contractility modulation (CCM) improved functional status and left ventricular ejection fraction whether implanted early or later. Implantation within 12 months after CCM‐eligible heart failure diagnosis might be associated with better survival, greater ejection‐fraction improvement, and fewer heart‐failure hospitalizations.

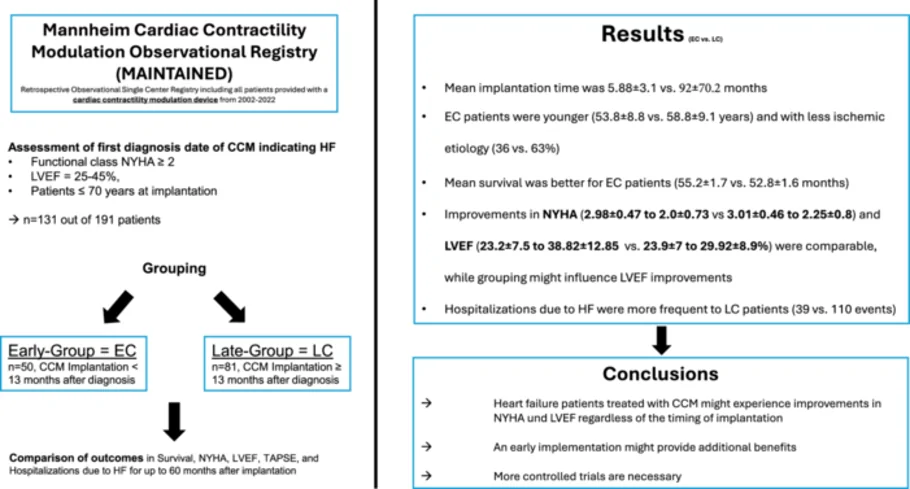

## 1 Introduction

Chronic Heart Failure (HF) remains an important cause of hospitalization, mortality and healthcare expenses. Despite improvements in therapy, patients with HF continuously experience severe limitations in different aspects of life [1, 2]. Hence, a greater emphasis on personalized HF therapy that addresses a broad range of modifiable disease aspects beyond survival alone, including functional improvements, electromechanical recovery, quality of life, frailty prevention, psycho‐cardiology and others [3, 4] has emerged. Apart from sodium‐glucose co‐transporter‐2 inhibitors (SGLT2), most established drugs for the treatment of HF with reduced ejection fraction (HFrEF) have depressant effects on the renin‐angiotensin‐aldosterone signaling pathways. Due to these and further side effects, patients' tolerance of guideline‐directed medical treatment (GDMT) remains a crucial limiting factor in immediately starting recommended drug combinations. Hence, a stepwise up‐titration rather than immediate combined initiation has most often been applied in clinical practice of HFrEF treatment, whereas a cautiously monitored initiation of all four class 1 recommended drugs at the earliest possible stage is now recommended for GDMT [5, 6]. However, symptomatic HFrEF with persistently reduced LVEF despite GDMT remains a challenge, and might additionally be addressed with cardiac implantable electrical devices (CIEDs) such as implantable cardioverter defibrillators (ICDs) or cardiac resynchronization therapy devices with ICD capabilities (CRT‐Ds) [3, 7, 8]. Both CIEDs provide protection from arrhythmic sudden cardiac death (SCD) for primary preventative reasons, while CRTs additionally aim for the reverse remodeling of structural HF impairment. However, before CIED implantations are carried out, several therapy‐relevant aspects should be considered, since therapy discontinuation for CIEDs is significantly more difficult than with drug treatment. In this respect, several previous trials have already evaluated the importance and relevance of implantation timing of CIEDs, as reflected in the guidelines. The timing of ICD and CRT‐D implantations are clearly defined, most often being provided for primary prevention reasons if 3 months of controlled GDMT has failed to reverse remodel the underlying HF [3, 8]. However, implantation timing is complicated further, as delaying therapy too much may not result in optimal or even beneficial outcomes, considering the clinical phenotype of “beyond the repair” HF [9, 10]. Cardiac contractility modulation (CCM) is an alternative device‐based therapy option for patients with a narrow QRS complex and symptomatic HF, and who have an LVEF ranging from 25% to 45% [11, 12, 13]. CCM has yet to be evaluated with respect to outcomes related to the timing of implantation.

## 2 Methods

### 2.1 Ethics Statement

The study was approved by the Ethics Committee of the Medical Faculty Mannheim, Heidelberg University (2020‐859‐AF11) and conducted in accordance with the Helsinki Declaration of 1975, as revised in 1983.

### 2.2 Study Population and Enrollment

The **MA**nnheim card**I**ac co**N**trac**T**ility modul**A**t**I**o**N** obs**E**rvational study (**MAINTAINED**) was designed as a retrospective observational single‐center registry, including all patients who were implanted with a CCM device at the University Medical Centre Mannheim between 2002 and 2022. Written informed consent was obtained from the patients involved. For the present analysis, the date of the first diagnosis of CCM indicating HF, which was defined as functional class NYHA ≥ 2 and LVEF = 25%–45%, was assessed in registry patients aged ≤ 70 years at implantation. Older patients and those without a clear retrospectively assessable diagnosis date were excluded from this approach. However, 3 months were added to that date to reflect guideline‐according medical treatment. The resulting date determined the earliest time at which CCM treatment might have been carried out. According to these criteria, a total of *n* = 131 patients from the registry were grouped into either Early‐ (EC, *n* = 50, CCM < 13 months after HF diagnosis) or Late‐Implantation Groups (LC, *n* = 81, CCM ≥ 13 months after HF diagnosis). Both groups were compared regarding survival, NYHA classification, intrinsic QRS‐width, LVEF, device associated complications, train delivery rate, hospitalizations due to HF and further parameters for up to 60 months after implantation.

### 2.3 Baseline and Follow‐Up Data

Baseline demographics as well as medication, echocardiographic parameters, and implantation procedure data were evaluated at baseline and at follow‐ups after one, three and 5 years. The patients were each fitted with the latest available generation of the CCM device. In both, group 1 (*n* = 40, 80%) and group 2 (n = 55, 68%), these were predominantly CCM generation III and IV devices. Follow‐up visits included interrogation of CCM‐ and ICD programming aiming to optimize both CCM delivery rate and ICD functions. Within this framework, optimized programming of the CCM device was carried out for each patient, according to criteria for optimizing CCM therapy [14]. During each follow‐up, the clinical evaluation included NYHA‐class, peak VO2 by cardiopulmonary exercise test, and the Minnesota Living with Heart Failure Questionnaire Score (MLWHFQ). Patients who expired during follow‐up were considered for statistical analysis (where necessary), as were patients whose CCM‐devices were explanted, or those who underwent heart transplantation during follow‐up. A broad range of CCM‐associated complications, such as lead‐infections or lead‐dysfunctions, were recorded separately. Echocardiographic examinations were performed using 2D measurements, M‐mode measurements and LVEF calculation by Simpson's method, including left ventricular end‐diastolic and ‐systolic diameters (LVEDD/LVESD), regional wall motion abnormalities, global longitudinal strain (GLS), right‐ and left atrium diameters, diastolic functions, tricuspid annular systolic velocity (TASV), tricuspid annular plane systolic excursion (TAPSE) and other measures. Blood samples were collected at each follow‐up including serum levels of potassium, creatinine, n‐terminal pro brain natriuretic peptide (NT‐pro‐BNP), hemoglobin, estimated glomerular filtration rate (eGFR), cholinesterase, internationalized normalized ratio of prothrombin time and others. Several HF‐related medications including angiotensin‐converting enzyme inhibitors (ACEI), angiotensin receptor blockers (ARB), angiotensin receptor‐neprilysin inhibitors (ARNI), aldosterone antagonists (AA), sodium‐glucose co‐transporter‐2 inhibitors (SGLT2), beta‐blockers (BB), amiodarone, ivabradine, different diuretics or cardiac glycosides were obtained from the medical reports.

### 2.4 Statistical Analysis

All statistical calculations were performed using SAS software, release 9.4 (Cary, USA), based on available data from the registry. For qualitative variables, absolute and relative frequencies are given. For quantitative and approximately normally distributed variables, mean values ± standard deviation (SD) were calculated. For skewed or ordinal data, minimum and maximum values are presented. The mean values of the two groups, EC versus LC, were compared using two‐sample *t*‐tests (in the case of normally distributed data) or the Mann‐Whitney U test. Survival curves were constructed using the Kaplan‐Meier method. A comparison of the Kaplan‐Meier curves was performed using the LogRank test. The logistic regression was performed as a multivariable model to verify the prediction of the MAGGIC‐score. MAGGIC‐score based predicted mortality was compared with observed mortality using the chi‐square test. To compare hospitalizations due to HF and anti‐tachycardia treatments, a chi‐square test was used and an odds ratio calculated. Baseline values were compared with 12‐month values with the paired *t*‐test (for normally distributed differences) or the Wilcoxon test (for non‐normally distributed differences). A two‐way repeated analysis of variance (rANOVA for the factors time and group) was performed to compare the mean values of distinct groups over the entire follow‐up period. Statistical significance was assumed for *p*‐values < 0.05.

## 3 Results

### 3.1 Baseline Characteristics

Registry patients aged ≤ 70 years were grouped into either the early CCM (EC, implantation < 13 months after the first diagnosis of CCM‐suitable HF, *n* = 50) or the late CCM (LC) group (implantation ≥ 13 months, *n* = 81). LC patients were older than EC patients (hereinafter EC values are given first and LC values second; 53.8 ± 8.8 vs. 58.8 ± 9.1 years, *p* = 0.04). Due to grouping criteria, the mean time to CCM implantation after the first diagnosis of CCM‐suitable HF differed significantly in both groups (5.85 ± 3.1 vs. 92.0 ± 70.2 months, *p* < 0.001). Furthermore, the time interval from previously implanted first CIED to CCM implementation differed (2.7 ± 2.9 vs. 41.8 ± 41.6 months, both *p* < 0.0001), with 11 of the previously implanted CIEDs being CRT devices (4 vs. 7, *p* > 0.05). However, the majority of previously implanted CIEDs were single‐chamber (24 vs. 42) or dual‐chamber ICDs (6 vs. 16, both *p* > 0.05) in both groups. LC patients suffered more from ischemic etiologies of HF than EC patients (36% vs. 63%, *p* = 0.003), while the distribution of dilated cardiomyopathy was similar in both groups (23 vs. 25, *p* > 0.05). Further differences were observed in history of smoking (49.3% vs. 70%, *p* = 0.02) and eGFR (69.4 ± 24.6 vs. 58.8 ± 22.7 mL/min/1.73m^2^; *p* = 0.016), whereas other cardiovascular risk factors such as diabetes, hypertension or dyslipidemia were similarly distributed in both groups. LC patients presented with a higher MAGGIC‐score (21.6 ± 5.1 vs. 24.1 ± 4.8, *p* = 0.008), while quality of life assessed with the MLWHFQ‐score was comparable (53.7 ± 21.7 vs. 44.9 ± 22.5, *p* = 0.14). Further parameters such as mean NYHA classification (3.0 ± 0.5 for both groups), the LVEF (23.2 ± 7.5% vs. 23.9 ± 7%), TAPSE (17.5 ± 7.1 mm vs. 17.1 ± 4.4 mm), intrinsic QRS‐width (107.8 ± 18.1 vs. 112.4 ± 20 ms) or NT‐pro‐BNP (3023.5 ± 3169 vs. 3949.3 ± 4300 ng/L) were equally distributed (each *p* > 0.05). Medical treatment differences were observed for thiazide diuretics (16% vs. 30.9%, *p* = 0.05) and AA (78% vs. 59.2%, *p* = 0.03). However, GDMT3, defined as a treatment combination including an ACEI/ARB/ARNI, a BB and an AA, was similar in both groups at the time of CCM implantation (74% vs. 60.5%, *p* = 0.11; see Table 1).

**TABLE 1:** Baseline characteristics for cardiac contractility modulation (CCM) recipients with either early‐ (EC, *n* = 50) or late (LC, *n* = 81) implantation after first diagnosis of CCM suitable heart failure.

	Total	EC	LC	p‐value (EC vs. LC)
Patients	131	50	81	
Age (years)	56.9 ± 9.3	53.8 ± 8.8	58.8 ± 9.1	**0.04**
Male	108 (82.4)	41 (82)	67 (82.7)	0.91
BMI (kg/m^2^)	30 ± 6.3	29.7 ± 6.2	30.2 ± 6.4	0.64
Hypertension	94 (24.8)	33 (68.7)	61 (80)	0.18
Obesity	58 (45.3)	18 (37.5)	40 (50)	0.17
Diabetes	49 (37.4)	14 (28)	35 (43.2)	0.08
Dyslipidemia	98 (75.4)	35 (71.4)	63 (77.8)	0.41
Smoking	75 (57.2)	35 (70)	40 (49.3)	**0.02**
Post‐HF (months)	59.2 ± 69.3	5.85 ± 3.1	92.0 ± 70.2	**< 0.0001**
Post‐CIED (months)	26.6 ± 37.7	2.7 ± 2.9	41.8 ± 41.6	**< 0.0001**
Post‐2013	76 (58)	29 (58)	47 (58)	0.55
MAGGIC‐Score	23.2 ± 5.1	21.6 ± 5.1	24.1 ± 4.8	**0.008**
MLWHFQ	50.5 ± 22.3	44.9 ± 22.5	53.7 ± 21.7	0.14
VO2max (mL/kg/min)	14 ± 3.9	15 ± 3.8	13.7 ± 3.8	0.14
NT‐pro‐BNP (ng/L)	4562 ± 3878	3023.5 ± 3169	3949.3 ± 4300	0.34
NYHA	3.0 ± 0.5	2.98 ± 0.47	3.01 ± 0.46	0.91
II	14 (10.7)	6 (12)	8 (9.8)	
III	103 (78.6)	38 (78)	64 (79.0)	
IV	14 (10.7)	5 (10)	9 (11.1)	
ICM	69 (52.7)	18 (36)	51 (63)	**0.003**
COPD	38 (29)	16 (32)	22 (27.2)	0.55
Creatinine (mg/dL)	1.4 ± 0.8	1.3 ± 0.9	1.5 ± 0.8	0.33
eGFR (mL/min/1.73 m^2^)	62.8 ± 23.9	69.4 ± 24.6	58.8 ± 22.7	**0.016**
Rhythm				
Atrial Fibrillation	25 (19.1)	6 (12)	19 (23.4)	0.10
QRS‐width (ms)	110.5 ± 19.3	107.8 ± 18.1	112.4 ± 20	0.19
VTA	30 (22.9)	5 (10)	25 (30.9)	**0.005**
ICD	127 (96.9)	48 (96)	79 (97.5)	0.62
Syncope	19 (16.2)	5 (10)	14 (20)	0.13
Echocardiography				
LVEF (%)	23.6 ± 7.2	23.2 ± 7.5	23.9 ± 7	0.59
LVEDD (mm)	65.7 ± 9.8	65.6 ± 10.2	65.7 ± 9.6	0.96
LVEDV (mL)	249.8 ± 106.2	261.5 ± 112.6	223.8 ± 96.7	0.50
TAPSE (mm)	17.3 ± 5.5	17.5 ± 7.1	17.1 ± 4.4	0.84
TRPG (mmHg)	41.6 ± 12.9	39.8 ± 9.2	42.8 ± 15.2	0.67
Medication				
BB	127 (96.9)	49 (98)	78 (96.3)	0.58
ACEI/ARB	103 (78.6)	42 (84)	61 (75.3)	0.23
ARNI	20 (15.3)	7 (14)	13 (16)	0.75
AA	87 (66.4)	39 (78)	48 (59.2)	**0.03**
Amiodarone	24 (18.6)	6 (12)	18 (22.8)	0.12
Cardiac Glycosides	19 (14.5)	5 (10)	14 (17.3)	0.25
Thiazide Diuretics	33 (25.2)	8 (16)	25 (30.9)	**0.05**
Loop Diuretics	109 (83.2)	43 (86)	66 (81.5)	0.50
SGLT2	4 (3.1)	2 (4)	2 (2.5)	0.64
GDMT3	86 (65.6)	37 (74)	49 (60.5)	0.11

*Note:* Values are given as mean ± SD or patient numbers (percentage). *p*‐values: group‐comparison EC versus LC at baseline (Chi‐Square). Statistical significance was assumed for *p*‐values < 0.05 (bold values). BMI = Body mass index, Post‐HF = Implantation time after first diagnosis of CCM indicating HF, Post‐CIED = Implantation time after implantation of a previous cardiac implantable electrical device, Post‐2013 = Proportion of implantation after the year 2013, MAGGIC‐Score = Meta‐analysis global group in HF mortality risk score, MLWHFQ = Minnesota Living with HF Questionnaire Score, VO2max = Maximal oxygen consumption during physical exertion, NT‐pro‐BNP = N‐terminal pro brain natriuretic peptide, NYHA = New York Heart Association classification, ICM = Ischaemic heart disease, COPD = Chronic obstructive pulmonary disease, eGFR = estimated glomerular filtration rate. VTA = History of ventricular tachyarrhythmias, ICD = Implantable cardioverter defibrillator (any). LVEF = Left ventricular ejection fraction, LV‐EDD/‐EDV = Left ventricular end‐diastolic diameter or volume, TAPSE = Tricuspid annular plane systolic excursion, TRPG = Tricuspid regurgitation peak pressure gradient. BB = Beta‐Blockers, ACEI = Angiotensin‐converting enzyme inhibitors, ARB = Angiotensin receptor blockers, ARNI = Angiotensin receptor‐neprilysin inhibitors, AA = Aldosterone antagonists, SGLT2 = Sodium‐glucose co‐transporter‐2 inhibitors, GDMT3 = Medical treatment combination including at least one ACEI/ARB/ARNI, BB and AA.

### 3.2 Therapy Safety and Efficiency, Disease Modification and Clinical Outcomes

Both EC and LC groups were compared for differences in outcomes for up to 60 months of follow‐up. Therapy safety, as assessed by the total complication rate, was comparable in both groups (49 vs. 99 events, *p* = 0.169). Therapy effectiveness, as assessed by mean delivery rates of CCM trains over the entire follow‐up, was better for EC patients (93.38 vs. 81.97%, *p* < 0.001). The observed mortality rates after 12 months (10.7% and 15%) and after 36 months (26.7% and 34.6%) were lower than those predicted by MAGGIC score for both groups. During 60 months of follow‐up, 13 EC patients (26%) and 36 LC patients (44.4%) died. The mean survival times were significantly better for the EC group (55.2 ± 1.7 vs. 52.8 ± 1.7 months, *p* = 0.03, Figure 1). During follow‐up, EC patients suffered fewer hospitalizations due to HF compared to the LC group (39 vs. 110 events, odds ratio 2.1, relative risk reduction 20.5%, *p* < 0.0001). There were no differences in hospitalizations for other reasons (91 vs. 144 events, odds ratio 1.36, *p* = 0.85) or in recorded events with ventricular tachycardias (46 vs. 85 events; odds ratio 1.79, *p* = 0.46) over the entire follow‐up (Figure 2). There was directional reduction in NT‐pro‐BNP levels in both groups, but these changes were not statistically significant (3023 ± 3169 to 925 ± 1453 vs. 3949 ± 4300 to 2847 ± 4072 ng/L after 60 months, both *p* > 0.05). Likewise, serum creatinine levels were stable for both groups during 60 months of follow‐up (1.3 ± 0.9 to 1.2 ± 0.28 vs. 1.5 ± 0.8 to 1.77 ± 1.22 mg/dL, both *p* > 0.05). Both groups revealed improvements in mean NYHA at each follow‐up (2.98 ± 0.47 to 2 ± 0.73 vs. 3.01 ± 0.46 to 2.25 ± 0.8 after 60 months, both *p* < 0.01, see Table 2).

**FIGURE 1:**
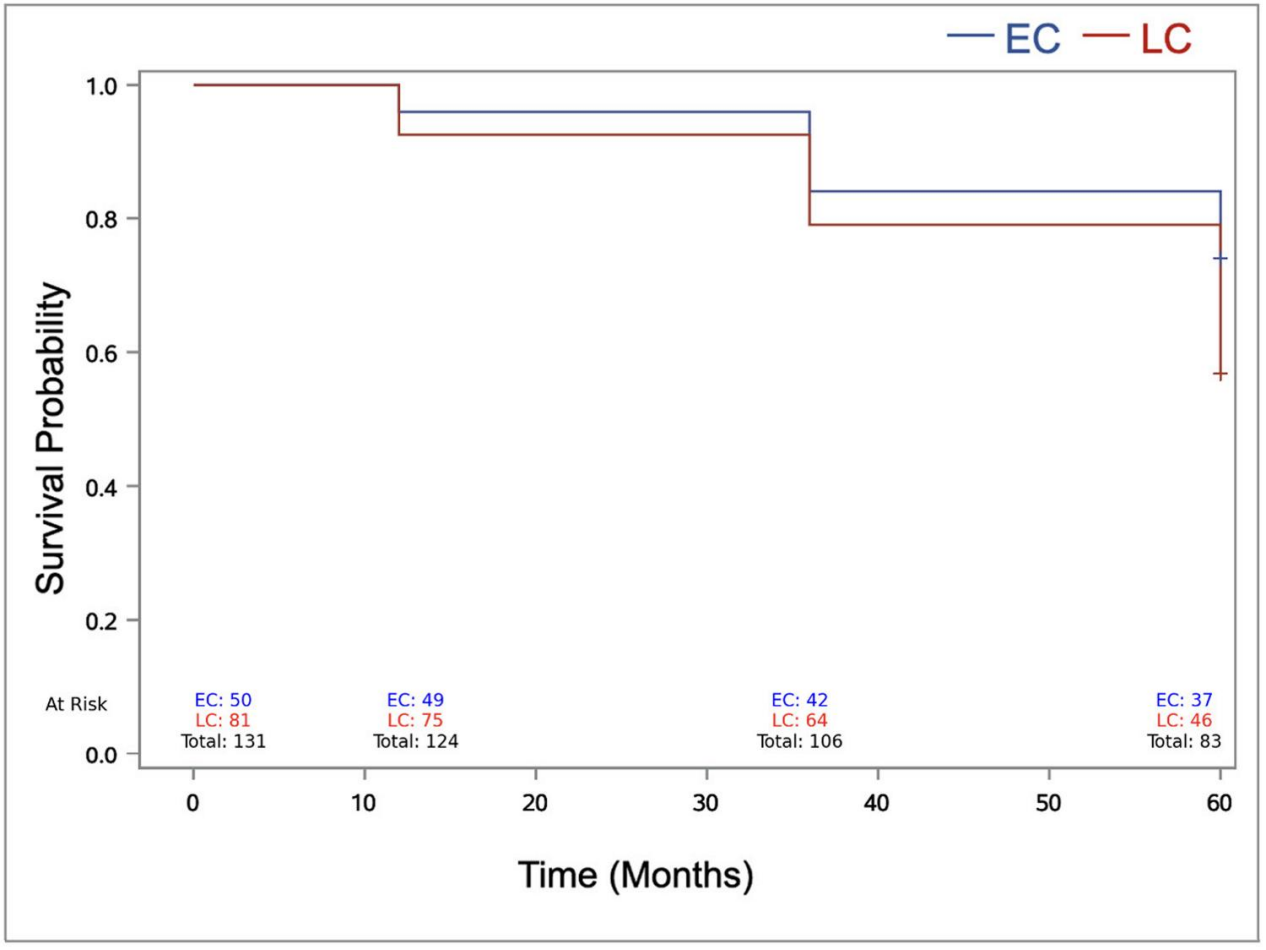
Kaplan‐Maier curves for cardiac contractility modulation (CCM) treated patients from the MAINTAINED registry. Based on implantation time after diagnosis of heart failure, the patients were grouped to either early‐ (EC, blue line) or late‐ (LC, red line) implantation status (< 13 months vs. ≥ 13 months after first diagnosis of CCM indicating heart failure). The number at risk for the total cohort and for the EC or LC groups are shown on the time line. Missing patients are due to uncompleted follow‐ups, heart transplantation and other reasons.

**FIGURE 2:**
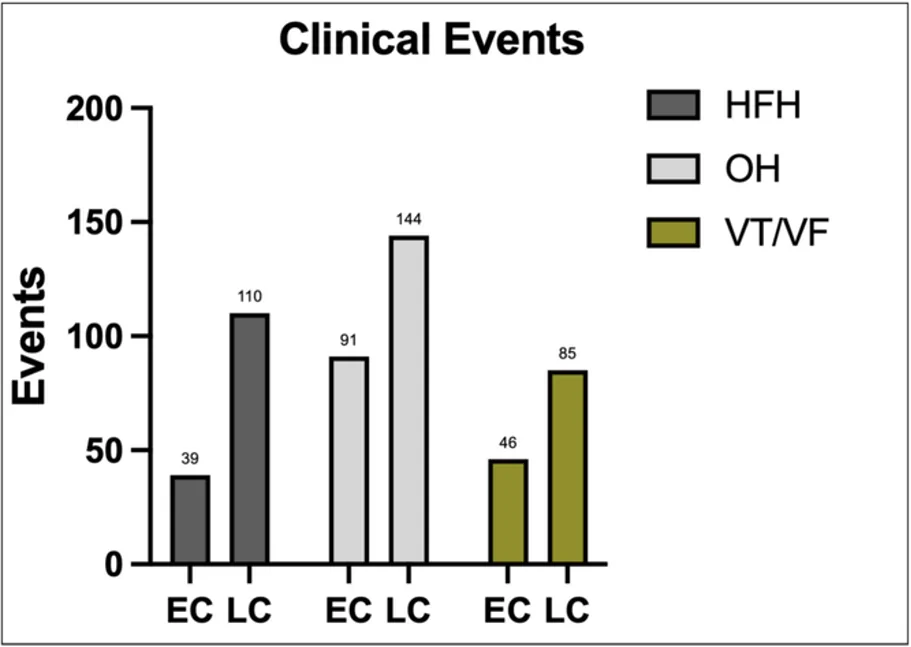
Illustration for distinct heart failure associated clinical events in cardiac contractility modulation (CCM) treated patients from the MAINTAINED registry, grouped to either early‐ (EC) or late‐(EC) implantation group (< 13 months vs. ≥ 13 months after first diagnosis of CCM indicating heart failure). Values are given as total number of events during entire 60 months of follow‐up. HFH = Heart failure hospitalizations, OH = Hospitalizations due to other reasons, VT/VF = sustained ventricular tachycardia or ventricular fibrillations, ATT = successful anti tachycardia treatments including anti tachycardia pacing and defibrillations.

**TABLE 2:** Alterations for several cardiac values in heart failure patients following treatment with cardiac contractility modulation (CCM) in either early‐ (EC) or late‐ (LC) implantation status (< 13 months vs. ≥ 13 months after first diagnosis of CCM indicating heart failure).

	Baseline		1FU		3FU		5FU		*p*‐values		
	EC	LC	EC	LC	EC	LC	EC	LC			
NYHA	2.98 ± 0.47	3.01 ± 0.46	2.34 ± 0.71	2.63 ± 0.64	2.3 ± 0.65	2.5 ± 0.66	2 ± 0.73	2.25 ± 0.8	**< 0.01**	**< 0.01**	0.06
NT‐pro‐BNP (ng/l)	3023 ± 3169	3949 ± 4300	2670 ± 6453	2976 ± 3393	1612 ± 2915	2332 ± 4628	925 ± 1453	2847 ± 4072	0.69	0.67	0.18
Creatinine (mg/dl)	1.3 ± 0.9	1.5 ± 0.8	1.5 ± 1.49	1.45 ± 0.61	1.46 ± 1.08	1.5 ± 0.65	1.2 ± 0.28	1.77 ± 1.22	0.58	0.21	0.26
LVEF (%)	23.2 ± 7.5	23.9 ± 7	34.1 ± 10.7	26 ± 7.8	36.8 ± 11.45	29.5 ± 7.4	38.8 ± 12.8	29.9 ± 8.9	**< 0.01**	**< 0.01**	**<0.01**
LVEDD (mm)	65.6 ± 10.2	65.7 ± 9.6	61 ± 8.1	66.6 ± 8.9	61.84 ± 7.6	66.4 ± 10.9	60.6 ± 9.6	65.3 ± 9.6	**< 0.01**	0.91	0.06
LVEDV (mL)	261 ± 112.6	223 ± 96.7	156 ± 64.6	321 ± 44.4	97.5 ± 34.6	251 ± 72	148.6 ± 25.8	146 ± 35.4	0.07	0.44	0.48
TAPSE (mm)	17.5 ± 7.1	17.1 ± 4.4	18.8 ± 4.9	17.7 ± 3.8	21.3 ± 5.1	16.5 ± 4.9	19.6 ± 5.1	18.9 ± 4.2	0.06	0.67	0.13
TASV (cm/s)	10.5 ± 2.1	8.75 ± 3.5	7.75 ± 2.87	8.8 ± 2.83	9 ± 2.83	7.5 ± 4.8	12.5 ± 2.65	9.75 ± 3.5	0.38	0.55	0.48
TRPG (mmHg)	39.8 ± 9.2	42.8 ± 15.2	23.7 ± 10.7	36.4 ± 12.6	28 ± 10.1	28.8 ± 6.7	26.3 ± 4.86	28 ± 5.1	**< 0.05**	0.39	0.13
QRS‐native (ms)	107.8 ± 18.1	112.4 ± 20	111.2 ± 19.9	113 ± 22.8	110.3 ± 21.7	117.9 ± 20.1	109.9 ± 21.4	112.7 ± 14.6	0.89	0.11	0.17

*Note*: Values are given as mean ± SD. *p*‐values (from left to right): baseline versus follow‐up comparison for EC, baseline versus follow‐up comparison for LC (t‐test, Wilcoxon‐Test), group comparison EC versus LC over entire 60 months of follow‐up (2‐Way‐rANOVA, factor grouping). Statistical significance was assumed for *p*‐values < 0.05 (bold values). NYHA = New York Heart Association classification, NT‐pro‐BNP = N‐terminal‐pro‐brain natriuretic peptide, LVEF = Left ventricular ejection fraction, LVEDD = Left ventricular end‐diastolic diameter, LVEDV = Left ventricular end‐diastolic volume, TAPSE = Tricuspid annular plane systolic excursion, TASV = Tricuspid annular systolic velocity, TRPG = Tricuspid regurgitation peak pressure gradient, QRS‐native = Width of the intrinsic QRS‐complex.

### 3.3 Electromechanical Reverse Remodeling

A broad range of electromechanical parameters was compared over the follow‐up period. While the LVEF increased at each follow‐up in both groups (23.2 ± 7.5% to 38.8 ± 12.8 vs. 23.9 ± 7 to 29.9 ± 8.9% after 60 months, both *p* < 0.01), there was a significant difference in the between‐group comparison favoring the EC group (two‐way ANOVA, factor groups, *p* < 0.01, see Figure 3 and Table 2). While there was a significant reduction in LVEDD in EC patients only (65.6 ± 10.2 to 60.6 ± 9.6 mm after 60 months, *p* < 0.01), further parameters of left ventricular reverse remodeling such as LVESV and LVEDV were not influenced in either group during follow‐up. Regarding right ventricular systolic function, there were no detectable improvements in either TAPSE (17.5 ± 7.1 to 19.6 ± 5.1 vs. 17.1 ± 4.4 to 18.9 ± 4.2 mm after 60 months, both *p* > 0.05, Figure 3) or TASV (8.7 ± 3.5 to 12.5 ± 2.6 vs. 8.75 ± 3.5 to 9.75 ± 3.5 cm/s after 60 months, both *p* > 0.05). However, tricuspid regurgitation peak gradient assessed estimation of pulmonary artery pressure (TRPG) decreased significantly only in EC patients (39.8 ± 9.2 to 26.3 ± 4.86 mmHg after 60 months, *p* < 0.05). The intrinsic QRS complex was stable without significant alterations for both groups (107.8 ± 18.1 to 109.9 ± 21.4 vs. 112.4 ± 20 to 112.7 ± 14.6 ms after 60 months, both *p* > 0.05, see Table 2).

**FIGURE 3:**
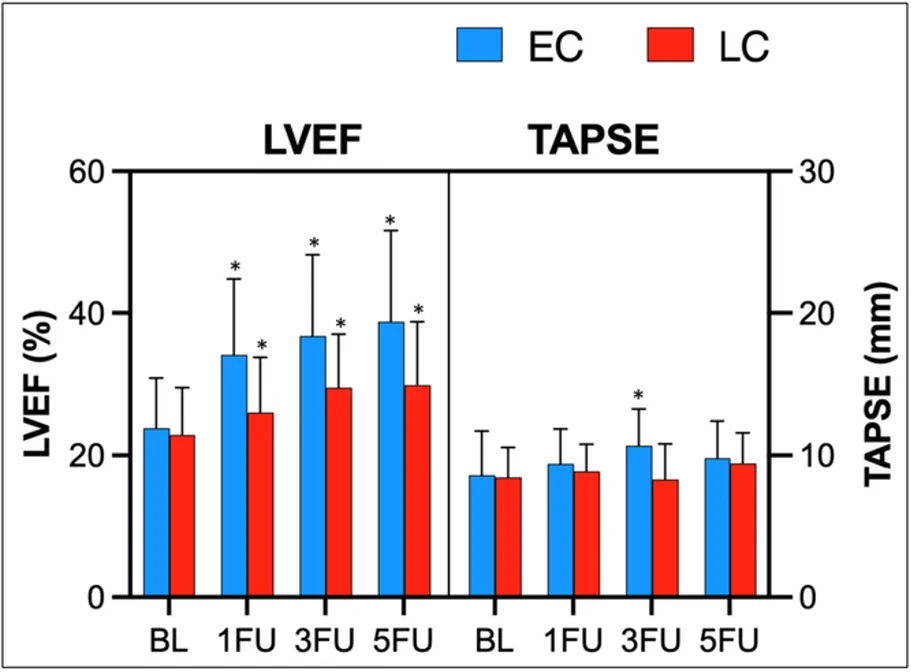
Illustration for the left ventricular ejection fraction (LVEF, biplane Simpsons method) and tricuspid annular plane systolic excursion (TAPSE, M‐mode) in cardiac contractility modulation (CCM) treated patients from the MAINTAINED registry, grouped to either advanced early‐ (EC, blue bars) or late‐ (LC, red bars) implantation group (< 13 months vs. ≥ 13 months after first diagnosis of CCM indicating heart failure). Values are given mean ± SD. p‐values: one‐way repeated analysis of variance, *:*p* < 0.05 compared to baseline. BL = Baseline, 1,3,5 FU = 12, 36 and 60 months follow‐up.

## 4 Discussion

Chronic heart failure remains a serious cause of hospitalization and death, despite improvements in medical and device therapy. Considering the increasing risk of cardiac death under failing heart conditions on the one hand, and the possible complications and adverse side effects on the other hand, the time frame of therapy implementation remains a crucial factor [15]. While the impact of timing and time‐dependent results has already been described for several drugs and CIEDs, we report here for the first time differences in outcomes between early and late implantation conditions following treatment with cardiac contractility modulation.

Consideration of CCM therapy is recommended for HF patients who remain symptomatic with an LVEF ranging from 25% to 45%. The positive inotropic and lusitropic effects of CCM trains, comparable to levosimendan‐induced enhancement of intracellular calcium metabolism, can be an additional therapy for these conditions, since no other medical or device therapy addresses similar pathways of the HF signaling cascade [16, 17, 18]. However, while optimized GDMT must be given in advance, the current guidelines do not further define or specify a certain time frame for CCM therapy implementation [3, 4]. As a chronic progressive syndrome, patients suffering from HF regularly present with a history of previously implanted ICDs for primary preventive reasons, and a prolonged history of medication changes, side‐effects, hospitalizations, interventional and structural treatments, arrhythmias, and further disease‐relevant events. In terms of ICDs, GDMT must be given for at least 3 months after the diagnosis of HF before an implantation for primary preventive reasons might be considered [3, 4, 19]. This postponement of implantation is driven by the desire to optimize LV function and to achieve an increase in LVEF to a range that obviates the need for ICD implantation, defined as HF with improved LVEF (HFimpEF) [20, 21]. Furthermore, device‐ and lead‐associated complications as well as the (potentially life‐long) risk of inappropriate shock delivery are considered in this context to avoid unnecessary ICD implantation [22]. Transvenous, subcutaneous and the newer substernal ICDs, provide anti‐tachycardia therapy options to protect against tachycardia associated sudden cardiac death [8]. However, only resynchronization or conduction system pacing devices are more likely able to revise pathophysiological pathways of HF, similar to drug treatments, by addressing interventricular and left intraventricular dyssynchrony [23, 24]. As such, only the latter devices can improve not only electromechanical parameters, but also clinical symptoms of HF. Therefore, HF patients should not expect further improvements in NYHA classification and biventricular systolic function following an ICD implantation for primary preventive reasons, but most certainly following CRT implantations. With respect to our study design on the timing of CCM implantation, several aspects of these clinical timelines must be considered. First, according to HF guidelines, the earliest date to provide CCM treatment would have been at least 3 months after the first diagnosis of HF. Secondly, this initial date of CCM‐suitable failing heart conditions does not necessarily must have been resulted in CCM therapy, especially considering HFimpEF conditions. Our retrospective analysis interestingly revealed a mean time to implantation in the late group of more than 7 years after the initial diagnosis, and more than 3 years after the first CIED implantation. Nevertheless, although some differences such as age or etiology‐distribution were observed, overall there were no further major differences seen. Both groups presented with comparable symptomatic HF with low LVEF values at the date of CCM implantation, despite this major gap of HF duration. Therefore, we were able to attest for the first time improvements in NYHA and LVEF, regardless of the timing of CCM implantation. Comparable positive effects on LVEF recovery in patients with HF persisting for more than 60 months were similarly reported for late implementation of ARNI therapy [25]. Compared to CRTs, a further distinction must be made between new implantations and upgrades, especially if indicated in the context of long‐lasting HF. Regular evaluation and, unfortunately, the disregard of QRS width, bundle branch block pattern, NYHA classification, and LVEF, even over extended periods, have led to the perception of undersupply of CRT devices, which undermines the value of highly specialized heart failure outpatient clinics [26, 27]. There are only limited data available comparing outcomes after CRT implantation in de novo HF versus long lasting HF [28]. However, meta‐analyses support the thesis that delayed CRT implantation is associated with more negative effects compared to earlier implantation [29]. The improved survival following early implantation and the higher hospitalization rate in LC patients in our study are consistent with these observations. However, it should be noted that the slightly longer survival time is of statistical rather than clinical significance. Although the improvements in LVEF, despite similar baseline values, were more pronounced in the EC group in terms of group comparison, LC patients also experienced improvements in NYHA class and LVEF, albeit not as pronounced. This in turn supports the assumption that a significant recovery of LV function and functional status is possible following CCM therapy, regardless of the duration of HF and the intermediate stage in which the patient survived long‐lasting HF [4, 15]. However, late treatment implementations are not uncommon [30]. Due to a lack of data, the patients' interim status during this nearly decade‐long period of living with chronic HF is unknown. They may have been in a stable NYHA I‐II condition, or might have even experienced improvements to HFimpEF, which in both cases would have rendered them ineligible for CCM therapy during this period. Reflecting further baseline values, this theory might additionally be supported, since most of the patients in both groups have been stated NYHA III. However, it remains questionable whether these patients remained in NYHA III status for so many years or whether they experienced HF progression after years of living with low‐symptomatic HFimpEF. Reflecting these considerations, an average duration of more than 60 months might not necessarily be interpreted as a delay in treatment. HF is known to have a long initial period of stability, while an increasingly rapid progression often only becomes apparent after years [4, 31]. In conclusion, these observations indicate that regular and thorough follow‐up of low‐symptomatic or HFImpEF patients, and the regular reevaluation of the current therapy are fundamentally important aspects in the management of HF. In late progressive stages, one should not shy away from considering CCM therapy. On the other hand, due to further study limitations and the points discussed above, such therapy decisions must be made with caution. Prospective and controlled data, likely including the upcoming new generation of CCM devices with ICD capability, could provide further insights in this regard.

## 5 Study Limitations

Several limitations must be acknowledged for this study. From a technical perspective, the study was designed as a retrospective observational registry, which cannot limit bias to the same extent as prospective data and randomized control trials. As GDMT developed over the years, the influence of newer medications, such as angiotensin‐neprilysin inhibitors (ARNI) or sodium‐glucose co‐transporter‐2 inhibitors (SGLT2), cannot be completely excluded. However, the SGLT2 influence is negligible, as they were more frequently prescribed after 2018. Furthermore, ARNIs were rarely prescribed in our study cohort (*n* = 20). Even if the utilization in both groups was comparable (Table 1), an influence on the results cannot be totally excluded. Additionally, the influence of further HF treatment options afterwards, such as mitral regurgitation repair, cannot be fully excluded. In general, since we have chosen more than 2 decades as the inclusion period, progress in medicine may have led to distortions in the results. In particular, the technical evolution of the device, such as the elimination of the atrial lead, must be taken into account when interpreting certain endpoints, even though this might have affected both groups simultaneously. Three further major limitations must be acknowledged for this approach. First, there is the very late implantation in the LC group. Due to a lack of data, it is unclear what clinical status the patients had during the time between diagnosis and implantation, why CCM therapy was not initiated sooner, and what ultimately led to the decision for late implantation. One solution to the variable and missing data could be introduction of standardized documentation forms for heart failure outpatient clinics, in which all possible therapies for patients are recorded during appointments and the reasons for and especially against initiating therapy are listed. Second, the unequal distribution of underlying heart failure etiology may also have contributed to the differences in outcomes. And finally, the timing of CCM implantation was not pre‐specified, making the comparison vulnerable to selection bias. Therefore, further controlled, multicenter, and prospective studies are needed to develop a precise algorithm for the timing of CCM implantation.

## 6 Conclusions

Heart failure patients might experience improvements in functional status and left ventricular reverse remodeling following CCM therapy, regardless of the timing of implantation. Early implementation of CCM within the first year of CCM suitable HF might provide additional benefits regarding survival, left ventricular systolic recovery and protection against heart failure hospitalizations.

## Author Contributions

Goekhan Yuecel designed the study and drafted the manuscript. Gamze Yildirim, Kristin Schreiner, Svetlana Hetjens, Konstantinos Giannakopoulos, Abbass Kodeih and Mostafa Khalil performed data acquiring and analysis. Babak Yazdani, Faeq Husain‐Syed, Daniel Duerschmied, William T. Abraham, Ibrahim Akin and Jürgen Kuschyk participated in study conception and design, interpretation of the data and revising the manuscript critically for important intellectual content.

## Funding

The authors have nothing to report.

## Conflicts of Interest

J.K. received speaker's fee from Impulse Dynamics Germany GmbH (former Stuttgart, now Frankfurt, Germany). The other authors report no relationships that could be construed as a conflict of interest.

## Data Availability

The data that support the findings of this study are available upon request from the corresponding author.
